# Supplementary material for: SNX8 enables lysosome reformation and reverses lysosomal storage disorder
Source: Nat Commun. 2024 Mar 22;15:2553. doi: 10.1038/s41467-024-46705-x (PMC10959956; doi:10.1038/s41467-024-46705-x)
Supplement: Supplementary file 1 — Supplementary Information [file 41467_2024_46705_MOESM1_ESM.pdf]

Fig. S1

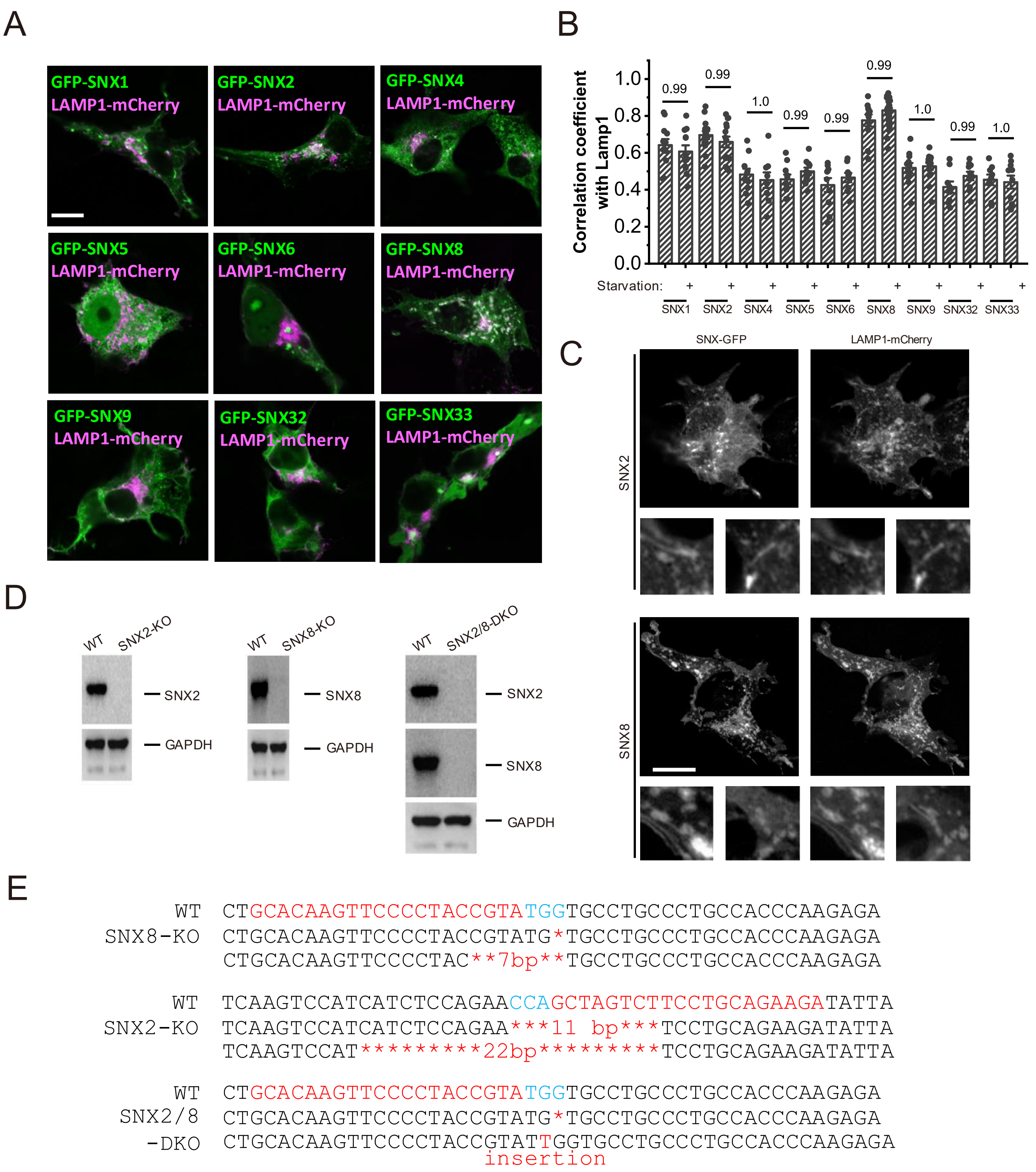

Supplementary Fig. 1. SNX2/8 participate in lysosome tubulation. A. Representative images of sample groups quantified in **Fig. 1B**. Scale bar = 10 μm. **B.** Quantification of the correlation coefficient between SNX-GFP proteins and LAMP1-mCherry in COS1 cells transfected with the listed SNX-GFP constructs for 24 h, and then treated with or without 6 h of serum starvation (n=12, 12, 15, 14, 10, 9, 10, 10, 10, 9, 11, 17, 11, 15, 9, 10, 9, 10, sequentially). **C**. Same cells shown in (**Fig. 1D**) were shown with separated channels. Scale bar = 10 μm. **D&E.** Western blot (**D**) and genomic sequencing (**E**) validation of SNX2 KO, SNX8 KO, and SNX2/8 DKO HeLa cells. For graphs, error bars are s.e.m, *: p<0.05, **: p<0.01, ***: p<0.001. Source data are provided as a Source Data file.

Fig. S2

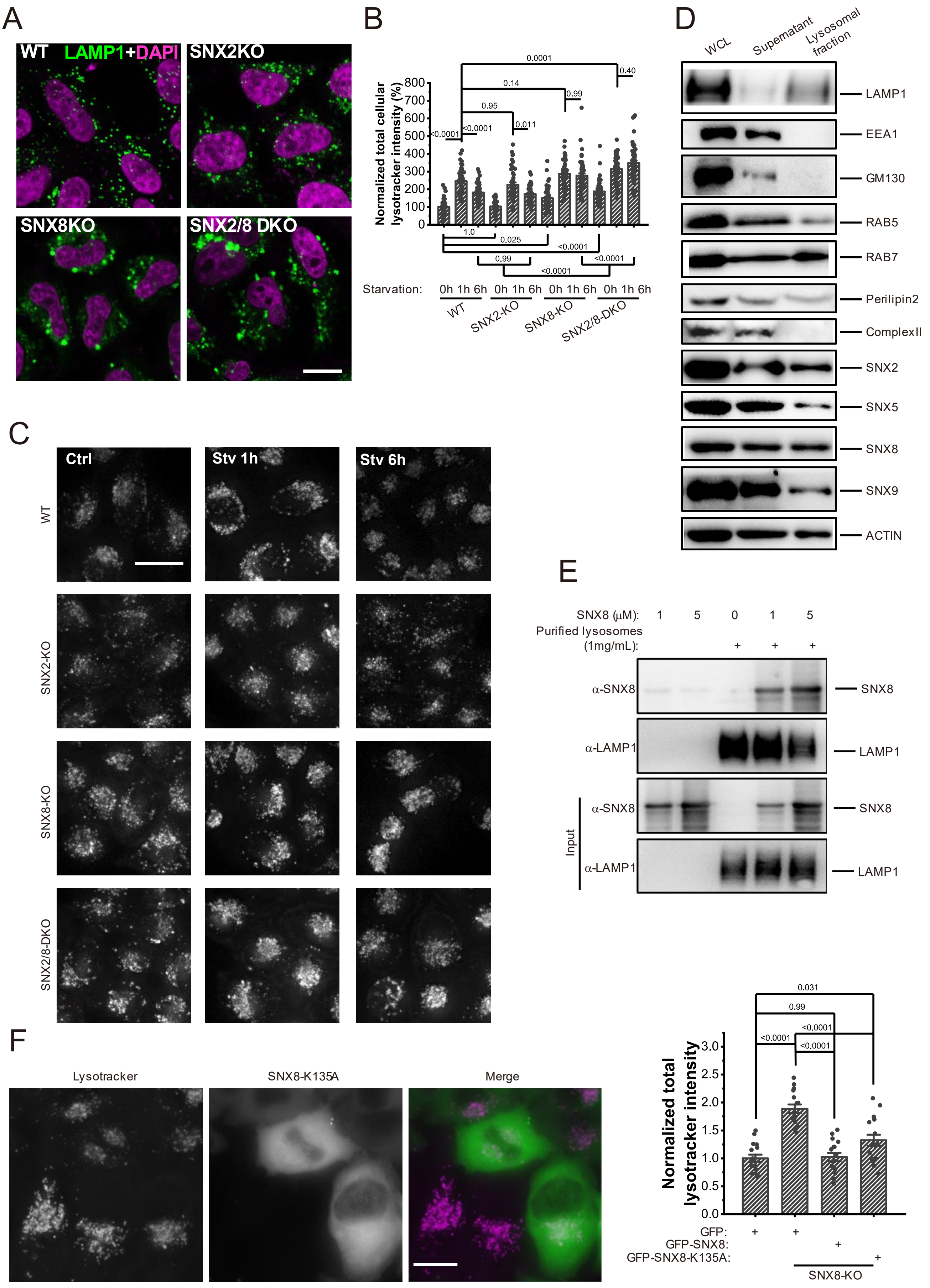
A
WT LAMP1+DAPI
SNX2KO
SNX8KO
SNX2/8 DKO
B
Normalized total cellular lysotracker intensity (%)
0
100
200
300
400
500
600
700
800
0.0001
0.40
0.14
0.99
0.95
0.011
<0.0001
<0.0001
1.0
0.025
<0.0001
0.99
<0.0001
<0.0001
Starvation: 0h 1h 6h 0h 1h 6h 0h 1h 6h 0h 1h 6h
WT
SNX2-KO
SNX8-KO
SNX2/8-DKO
C
Ctrl
Stv 1h
Stv 6h
WT
SNX2-KO
SNX8-KO
SNX2/8-DKO
D
WCL
Supernatant
Lysosomal fraction
LAMP1
EEA1
GM130
RAB5
RAB7
Perilipin2
ComplexII
SNX2
SNX5
SNX8
SNX9
ACTIN
E
SNX8 (μM): 1 5 0 1 5
Purified lysosomes (1mg/mL): + + +
α-SNX8
α-LAMP1
α-SNX8
α-LAMP1
Input
SNX8
LAMP1
SNX8
LAMP1
F
Lysotracker
SNX8-K135A
Merge
Normalized total lysotracker intensity
0.0
0.5
1.0
1.5
2.0
2.5
3.0
0.031
0.99
<0.0001
<0.0001
<0.0001
GFP: + +
GFP-SNX8: +
GFP-SNX8-K135A: +
SNX8-KO

Fig. S2 cont'd

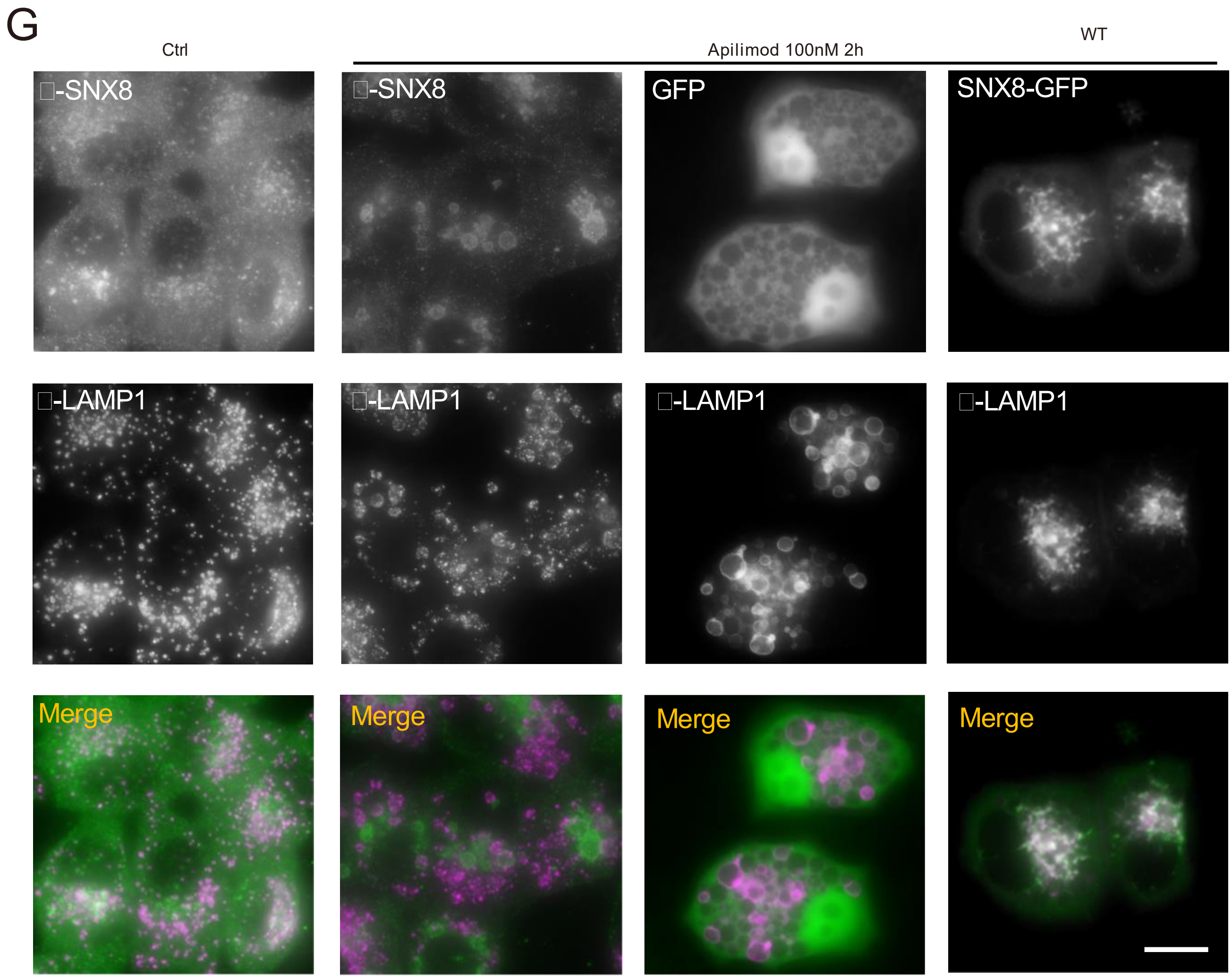

Supplementary Fig. 2. Loss of SNX8 causes enlarged lysosomes. A. Representative immunostaining images of endogenous LAMP1 in WT, SNX2 KO, SNX8 KO, and SNX2/8 DKO HeLa cells. Scale bar = 10 μm. **B&C.** Quantifications (**B**) and representative images (**C**) of lysotracker intensity in WT, SNX2 KO, SNX8 KO, and SNX2/8 DKO HeLa cells treated with 1 h or 6 h of starvation or left untreated (n=50 for all groups). **D**. Supernatant and purified pellet from the lysosome purification protocol (see Methods) were analyzed for the organellar markers and SNXs labeled by the blots. **E.** SFB-SNX8 and lysosomes were separately purified from HEK 293T cells and *SNX8 KO* HeLa cells, respectively, then mixed at indicated concentration in 150 mM NaCl, 20 mM HEPES (pH=7.4) for 20 min, pelleted and washed for three times and blotted. **F.** WT and SNX8-KO HeLa cells were transfected with constructs indicated in the plot for 24 h, and then stained with lysotracker and quantified (n=16 for all groups). Representative images of SNX8-KO cells transfected with SNX8-K135A were shown to the left. **G**. WT HeLa cells were untransfected or transfected with GFP/SNX8-GFP for 24 h, and then treated with 1 μM apilimod for 2 h, then fixed and stained with anti-Lamp1 (and anti-SNX8 for untransfected groups) antibodies. Representative images of sample groups were shown. Scale bar = 10 μm. For graphs, error bars are s.e.m, *: p<0.05, **: p<0.01, ***: p<0.001. Source data are provided as a Source Data file.

Fig. S3

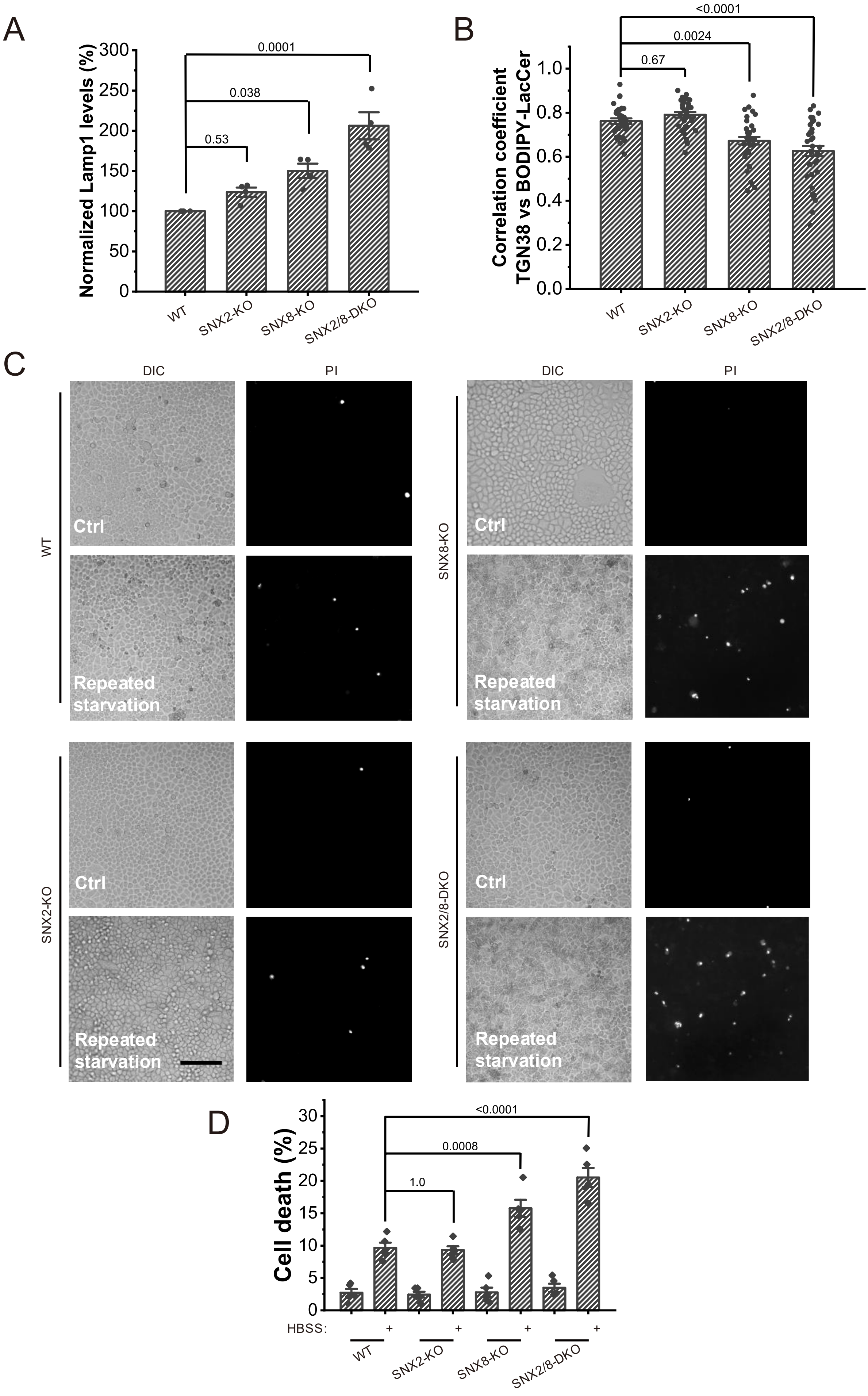

A
Normalized Lamp1 levels (%)
300
250
200
150
100
50
0
0.0001
0.038
0.53
WT
SNX2-KO
SNX8-KO
SNX2/8-DKO
B
Correlation coefficient
TGN38 vs BODIPY-LacCer
1.0
0.8
0.6
0.4
0.2
0.0
<0.0001
0.0024
0.67
WT
SNX2-KO
SNX8-KO
SNX2/8-DKO
C
DIC
PI
WT
SNX8-KO
SNX2-KO
SNX2/8-DKO
Ctrl
Repeated starvation
D
Cell death (%)
30
25
20
15
10
5
0
<0.0001
0.0008
1.0
HBSS:
+
WT
SNX2-KO
SNX8-KO
SNX2/8-DKO

Supplementary Fig. 3. Loss of SNX8 leads to LSD-like phenotypes. A. Quantifications of Western blot results as shown in (**Fig. 3A**) (n=4 for all groups). **B.** Quantifications of sample groups as shown in (**Fig. 3D**) (n=33, 32, 35, 37, sequentially). **C.** Representative images of sample groups quantified in (**Fig. 3F**). **D**. WT, SNX2 KO, SNX8 KO, and SNX2/8 DKO HeLa cells were incubated with HBSS for 8 h and cell death was assayed with the LDH release assay. (n=5 for all groups). Scale bar = 200 μm. For graphs, error bars are s.e.m, *: $p<0.05$, **: $p<0.01$, ***: $p<0.001$. Source data are provided as a Source Data file.

Fig. S4

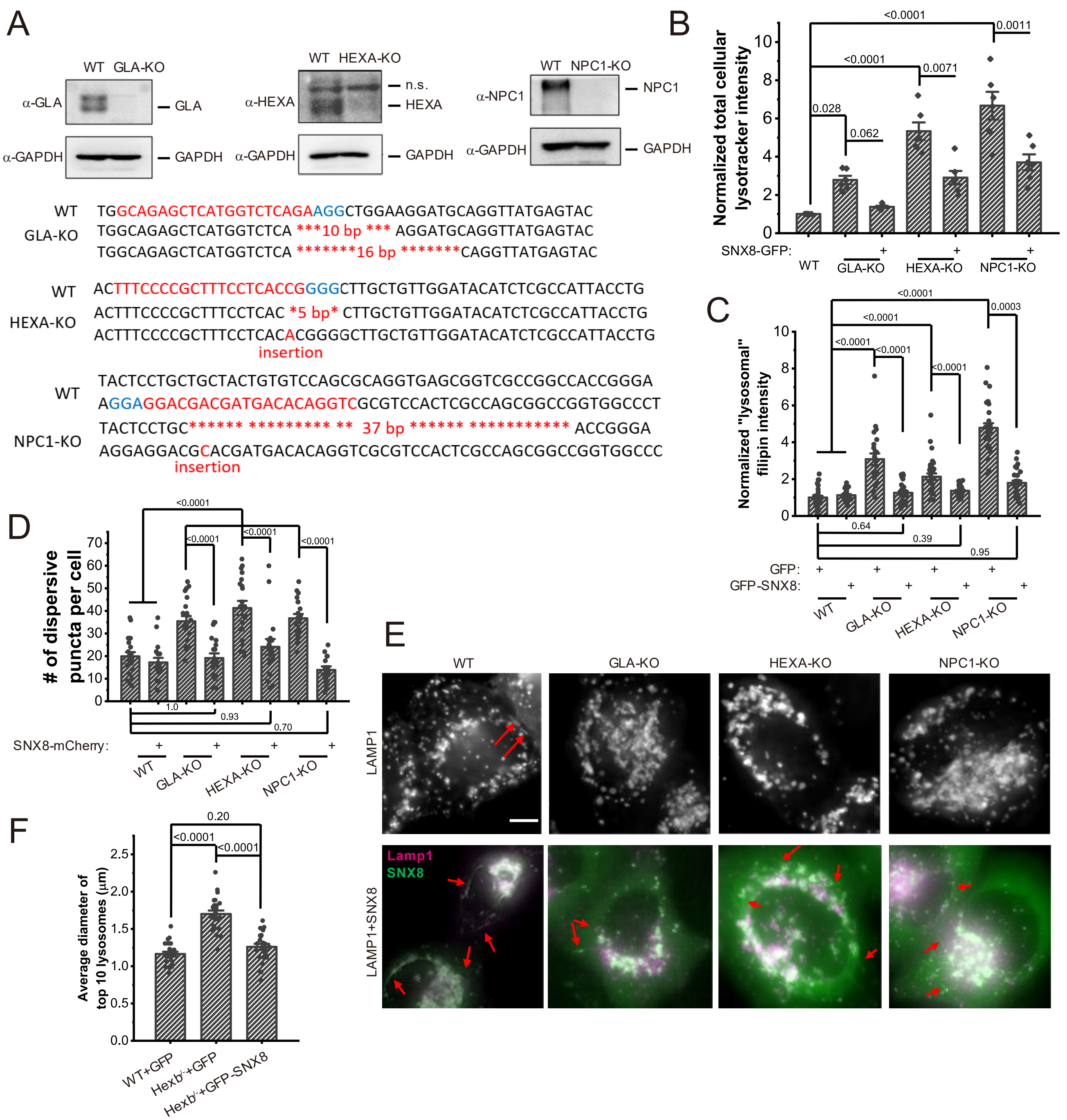

Supplementary Fig. 4. Overexpression of SNX8 rescues LSD phenotypes in LSD model cells. A. Western blot and genomic sequencing confirmation of GLA KO, HEXA KO, and NPC1 KO HeLa cells. **B.** WT and LSD model HeLa cells were untransfected or transfected with SNX8-GFP for 24 h, and then stained with lysotracker for 1 h before subjected to flow cytometry analysis of total cellular lysotracker. (n=5 for all groups). **C.** Quantifications of sample groups are shown in (**Fig. 4C**) (n=30 for all groups). **D.** Quantifications of sample groups are shown in (**Fig. 4D**) (n=20, 17, 20, 20, 20, 17, 20, 15, sequentially). **E**. Representative images of sample groups shown in (**Fig. 4E**). **F.** Quantification of the diameter of top 10 largest lysosomes for cell groups shown in (**Fig. 5A**) (n=25 for all groups). Scale bar = 5 μm. For graphs, error bars are s.e.m, *: p<0.05, **: p<0.01, ***: p<0.001. Source data are provided as a Source Data file.

Fig. S5

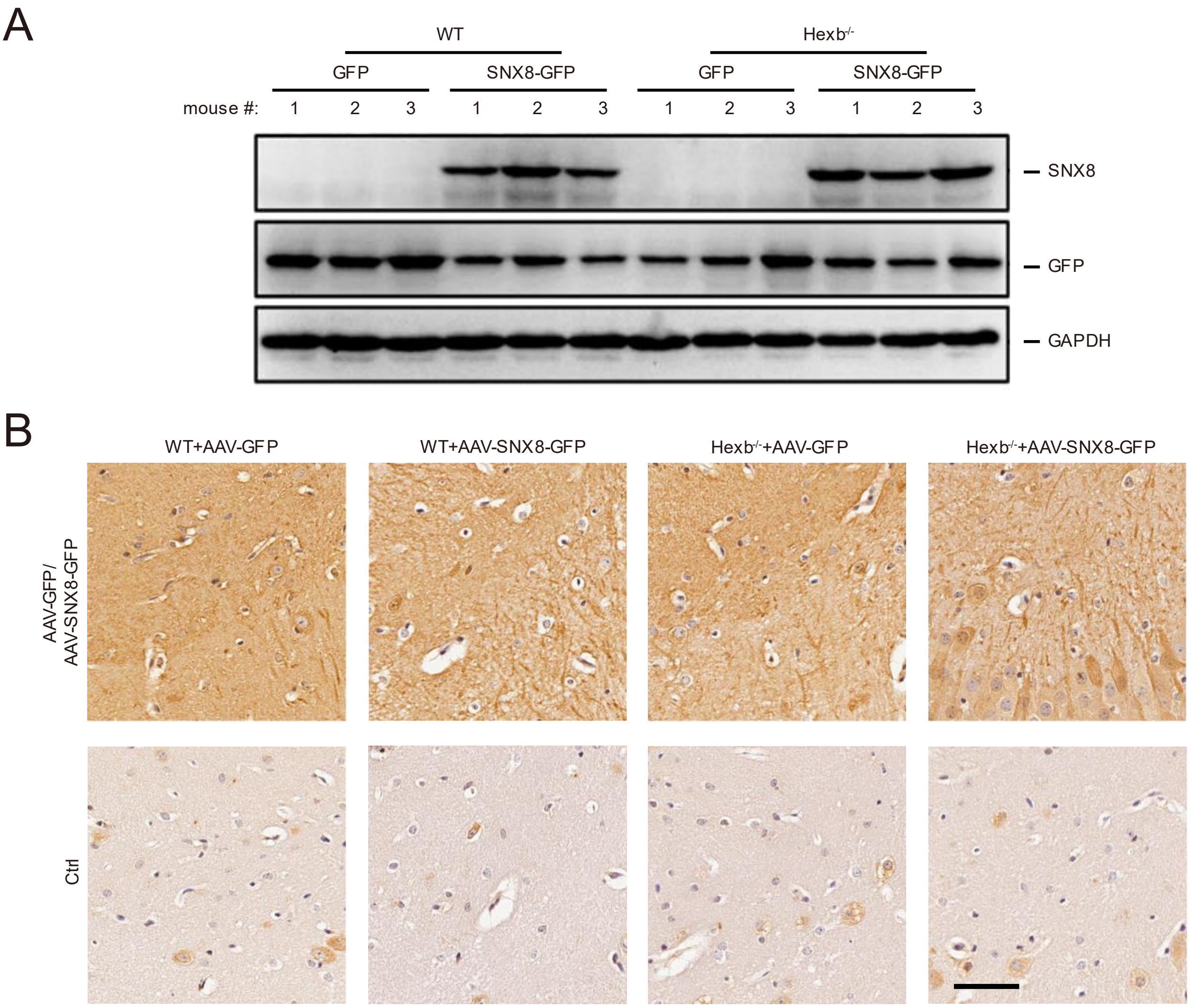

Supplementary Fig. 5. Confirmation of AAV-GFP and AAV-SNX8-GFP expression in mouse brains. A. AAV-SNX8 or AAV-GFP was delivered to mouse brain as described in the Methods, and the expression was examined through Western blots at 3 months of age (GFP-P2A-SNX8-FLAG is cleaved into GFP and SNX8-FLAG after translation). B. Immunohistochemistry of mouse brain sections (WT/Hexb$^{-/-}$ mice injected with AAV-SNX8 or AAV-GFP versus their control littermates) using anti-GFP antibody. Scale bar = 100 μm. Source data are provided as a Source Data file.

Fig. S6

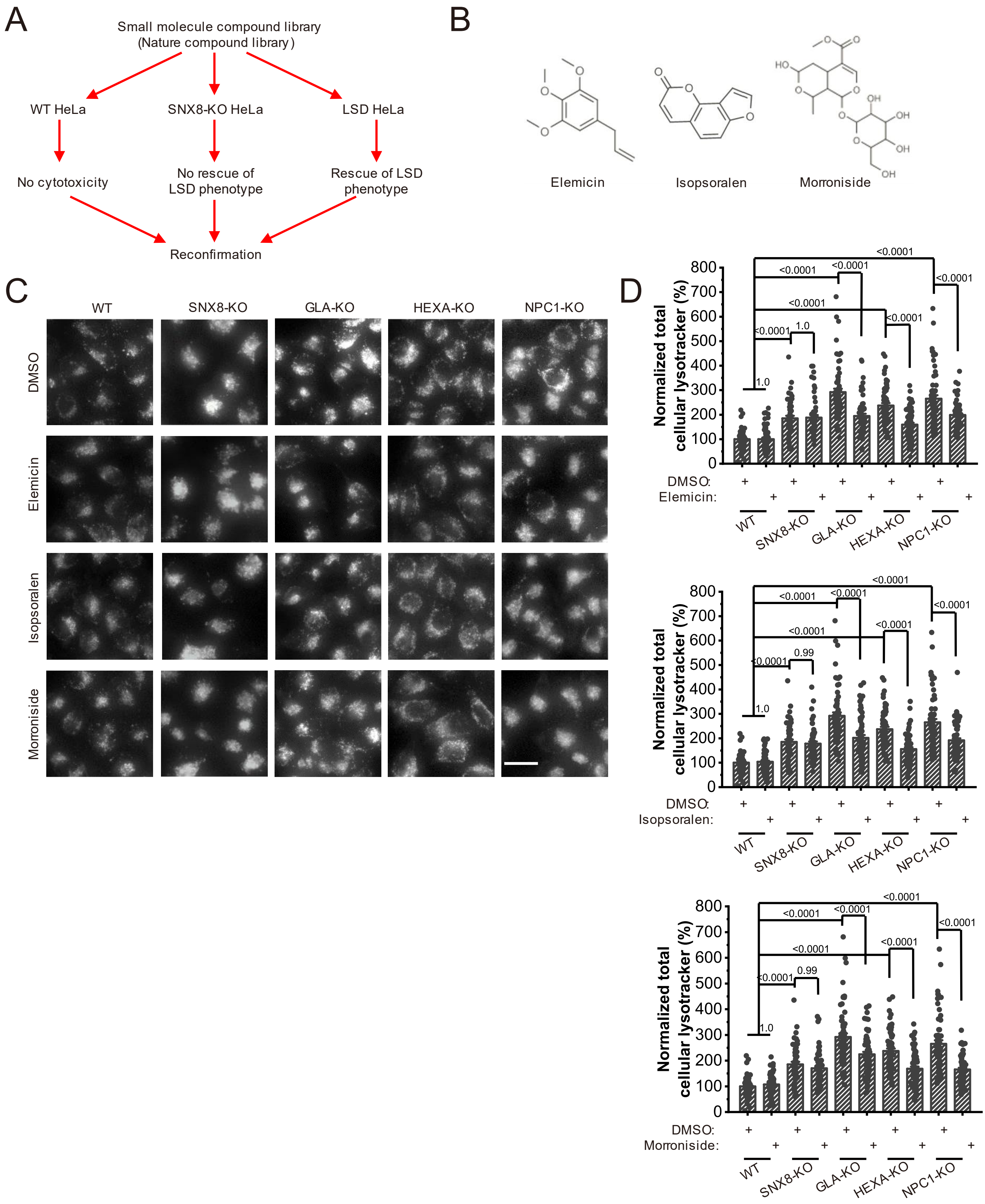
A
Small molecule compound library
(Nature compound library)
WT HeLa
SNX8-KO HeLa
LSD HeLa
No cytotoxicity
No rescue of
LSD phenotype
Rescue of LSD
phenotype
Reconfirmation
B
Elemicin
Isopsoralen
Morroniside
C
WT
SNX8-KO
GLA-KO
HEXA-KO
NPC1-KO
DMSO
Elemicin
Isopsoralen
Morroniside
D
Normalized total
cellular lysotracker (%)
DMSO:
Elemicin:
Isopsoralen:
Morroniside:
WT
SNX8-KO
GLA-KO
HEXA-KO
NPC1-KO

Fig. S6 cont'd

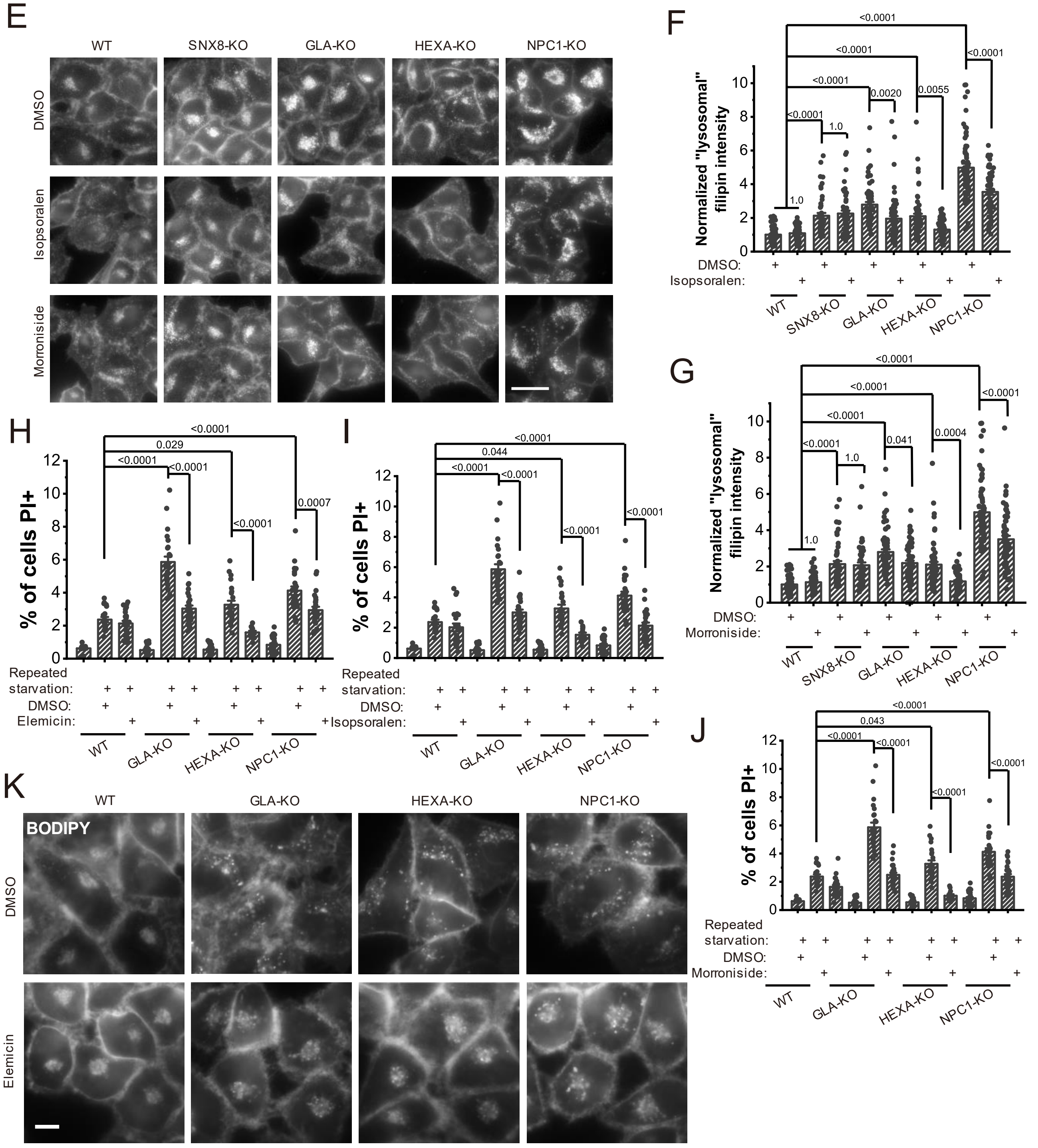
E
WT
SNX8-KO
GLA-KO
HEXA-KO
NPC1-KO
DMSO
Isopsoralen
Morroniside
F
Normalized "lysosomal" filipin intensity
DMSO:
Isopsoralen:
WT
SNX8-KO
GLA-KO
HEXA-KO
NPC1-KO
<0.0001
1.0
0.0020
0.0055
G
Normalized "lysosomal" filipin intensity
Morroniside:
0.041
0.0004
H
% of cells PI+
Repeated starvation:
Elemicin:
0.029
0.0007
I
0.044
J
0.043
K
BODIPY

Supplementary Fig. 6. Small molecule drugs suppress LSD phenotypes in LSD model cells. A. Flow chart of the screen to identify small molecules that may rescue LSD through SNX8. **B.** molecular structures of the three small molecules identified in the screen. Scale bar = 20 μm. **C&D.** WT, SNX8 KO, GLA KO, HEXA KO, and NPC1 KO HeLa cells were treated with DMSO, Elemicin (20 μM), Isopsoralen (20 μM), or Morroniside (20 μM) for 24 h and then stained with lysotracker. Representative images are shown in (**C**), and quantifications are shown in (**D**) (n=54 for SNX8-KO cells and 64 for all other cells). Scale bar = 20 μm. **E-G.** WT, SNX8 KO, GLA KO, HEXA KO, and NPC1 KO HeLa cells were treated with DMSO, Isopsoralen (20 μM) or Morroniside (20 μM) for 24 h, and then stained with filipin to visualize intracellular free cholesterol. Representative images are shown in (**E**), and quantifications are shown in (**F**) for Isopsoralen and (**G**) for Morroniside (n=58 for SNX8-KO cells and 64 for all other cells). Scale bar = 20 μm. **H-J.** WT, SNX8 KO, GLA KO, HEXA KO, and NPC1 KO HeLa cells were subjected to a repeated *serum starvation* protocol with 12 h starvation followed by 12 h complete medium, repeated 3 times, and with the co-application of DMSO, Elemicin (20 μM, **H**, n=12, 20, 24, 23, 24, 24, 26, 23, 12, 28, 24, 24, sequentially), Isopsoralen (20 μM, **I**, n=12, 20, 22, 23, 24, 26, 26, 23, 12, 28, 24, 24, sequentially), or Morroniside (20 μM, **J**, n=12, 20, 24, 23, 24, 24, 26, 23, 12, 28, 24, 24, sequentially). Cells were then stained with PI to visualize dead cells. **K**. Representative images for sample groups quantified in **Fig. 6E**. Scale bar = 10 μm. For graphs, error bars are s.e.m, *: $p<0.05$, **: $p<0.01$, ***: $p<0.001$. Source data are provided as a Source Data file.

Fig. S7

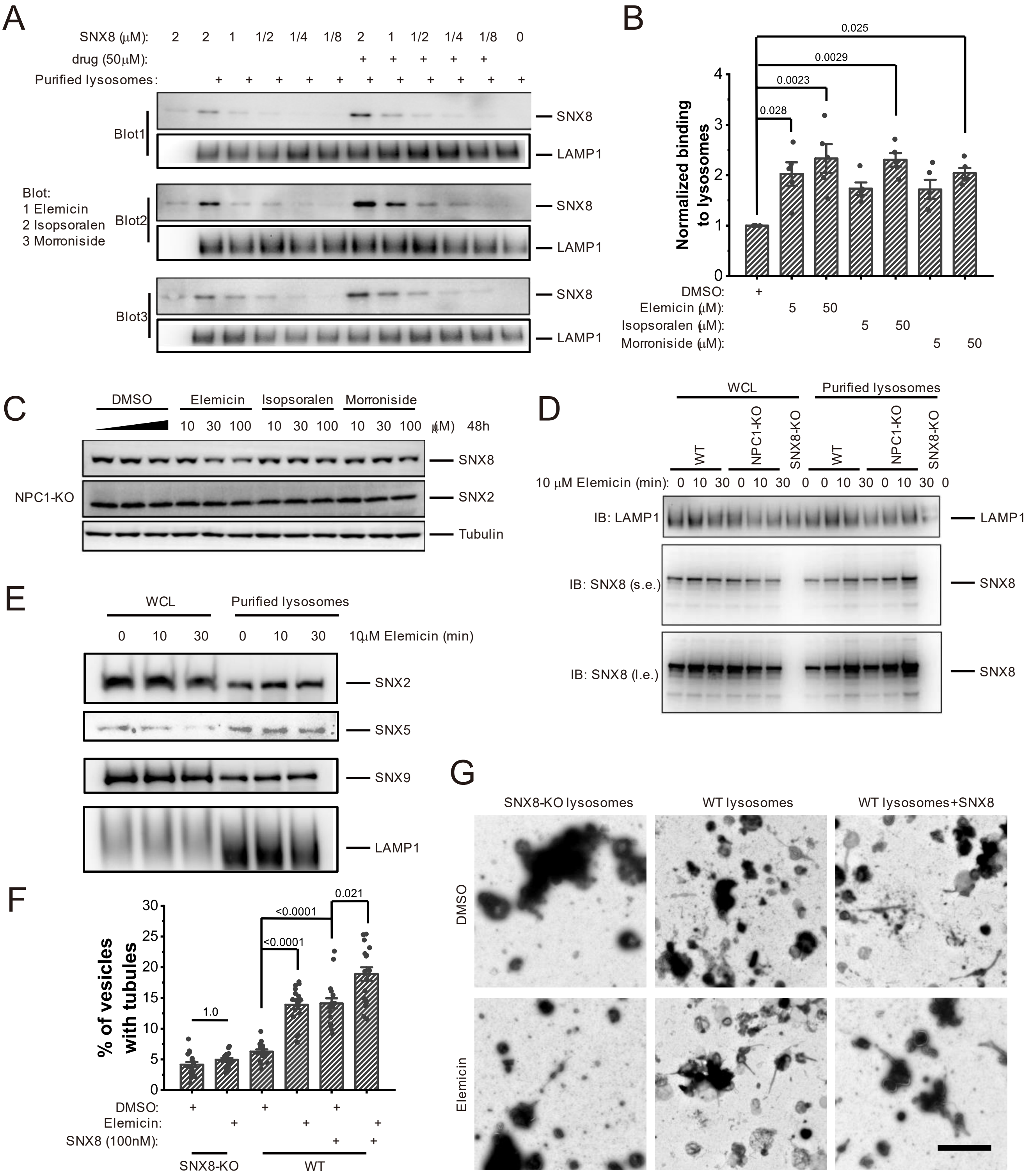

A
SNX8 (μM): 2 2 1 1/2 1/4 1/8 2 1 1/2 1/4 1/8 0
drug (50μM): + + + + +
Purified lysosomes: + + + + + + + + + + +
Blot1
Blot2
Blot3
SNX8
LAMP1
Blot:
1 Elemicin
2 Isopsoralen
3 Morroniside
B
Normalized binding to lysosomes
0.025
0.0029
0.0023
0.028
DMSO: +
Elemicin (μM): 5 50
Isopsoralen (μM): 5 50
Morroniside (μM): 5 50
C
DMSO
Elemicin
Isopsoralen
Morroniside
10 30 100 10 30 100 10 30 100 (μM) 48h
NPC1-KO
SNX8
SNX2
Tubulin
D
WCL
Purified lysosomes
WT
NPC1-KO
SNX8-KO
10 μM Elemicin (min): 0 10 30 0 10 30 0 0 10 30 0 10 30 0
IB: LAMP1
IB: SNX8 (s.e.)
IB: SNX8 (l.e.)
LAMP1
SNX8
E
WCL
Purified lysosomes
0 10 30 0 10 30 10μM Elemicin (min)
SNX2
SNX5
SNX9
LAMP1
F
% of vesicles with tubules
1.0
<0.0001
<0.0001
0.021
DMSO: + + +
Elemicin: + + +
SNX8 (100nM): + +
SNX8-KO
WT
G
SNX8-KO lysosomes
WT lysosomes
WT lysosomes+SNX8
DMSO
Elemicin

Supplementary Fig. 7. Small molecule drugs enhance the binding of SNX8 with lysosomes. A&B. Purified lysosomes from HeLa cells at 1 mg/mL were incubated with purified SNX8 at indicated concentrations, with DMSO or with each of the small molecules at 50 μM for 20 min in 150 mM NaCl, 20 mM HEPES (pH=7.4). Then, the mixture was pelleted and washed three times and blotted against SNX8 (**A**). Band intensity was measured with ImageJ and quantified as shown in (**B**) (n=5 for all groups, normalized to the DMSO control for each repeat). **C.** NPC1 KO HeLa cells were treated with DMSO or each of the three molecules at the indicated concentration for 48 h, and expression levels of SNX2 and SNX8 were examined by Western blots. **D.** WT and NPC1 KO HeLa cells were treated with 10 μM Elemicin for the indicated time, and lysosomes were then purified and blotted for lysosomal SNX8, with SNX8 KO cells as a negative control. E. Lysosomes and whole cell lysates purified from NPC1 KO cells same as those used in (D.) were blotted against SNX2, SNX5, and SNX9. No apparent changes in lysosomal localization of these sorting nexins was observed. **F&G**. Quantifications (**F**) (n=18 fields for all groups) and representative images (**G**) of purified lysosomes imaged with SEM to assess in vitro tubulation. Lysosomes were purified from SNX8 KO or WT HeLa cells as described in the Methods, then treated with DMSO or 100 μM Elemicin or with the addition of 100 nM SNX8 as indicated. The mixture was incubated for 20 min at room temperature and then stained with 2% uranyl acetate and examined with SEM. Scale bar = 2 μm. For graphs, error bars are s.e.m, *: $p<0.05$, **: $p<0.01$, ***: $p<0.001$. Source data are provided as a Source Data file.
