# Supplementary material for: SNX8 enables lysosome reformation and reverses lysosomal storage disorder
Source: Nat Commun. 2024 Mar 22;15:2553. doi: 10.1038/s41467-024-46705-x (PMC10959956; doi:10.1038/s41467-024-46705-x)
Supplement: Supplementary file 3 — Reporting Summary [file 41467_2024_46705_MOESM3_ESM.pdf]

Corresponding author(s): Xin-Hua Feng

Last updated by author(s): Dec 21, 2023

Reporting Summary

Nature Portfolio wishes to improve the reproducibility of the work that we publish. This form provides structure for consistency and transparency in reporting. For further information on Nature Portfolio policies, see our Editorial Policies and the Editorial Policy Checklist.

Statistics

For all statistical analyses, confirm that the following items are present in the figure legend, table legend, main text, or Methods section.

n/a	Confirmed	
☐	☒	The exact sample size (*n*) for each experimental group/condition, given as a discrete number and unit of measurement
☐	☒	A statement on whether measurements were taken from distinct samples or whether the same sample was measured repeatedly
☐	☒	The statistical test(s) used AND whether they are one- or two-sided<br>*Only common tests should be described solely by name; describe more complex techniques in the Methods section.*
☐	☒	A description of all covariates tested
☒	☐	A description of any assumptions or corrections, such as tests of normality and adjustment for multiple comparisons
☐	☒	A full description of the statistical parameters including central tendency (e.g. means) or other basic estimates (e.g. regression coefficient) AND variation (e.g. standard deviation) or associated estimates of uncertainty (e.g. confidence intervals)
☐	☒	For null hypothesis testing, the test statistic (e.g. *F*, *t*, *r*) with confidence intervals, effect sizes, degrees of freedom and *P* value noted<br>*Give P values as exact values whenever suitable.*
☒	☐	For Bayesian analysis, information on the choice of priors and Markov chain Monte Carlo settings
☒	☐	For hierarchical and complex designs, identification of the appropriate level for tests and full reporting of outcomes
☒	☐	Estimates of effect sizes (e.g. Cohen's *d*, Pearson's *r*), indicating how they were calculated

Our web collection on statistics for biologists contains articles on many of the points above.

Software and code

Policy information about availability of computer code

Data collection	No software was used
Data analysis	Softwares used include ImageJ for image analysis, Microsoft Excel and Origin 2022 for statistical analysis

For manuscripts utilizing custom algorithms or software that are central to the research but not yet described in published literature, software must be made available to editors and reviewers. We strongly encourage code deposition in a community repository (e.g. GitHub). See the Nature Portfolio guidelines for submitting code & software for further information.

Data

Policy information about availability of data

All manuscripts must include a data availability statement. This statement should provide the following information, where applicable:

- Accession codes, unique identifiers, or web links for publicly available datasets
- A description of any restrictions on data availability
- For clinical datasets or third party data, please ensure that the statement adheres to our policy

The datasets generated during and/or analysed during the current study are included in the manuscript, Supplementary information, and Source Data file. Data availability statement is included in the manuscript.

Human research participants

Policy information about studies involving human research participants and Sex and Gender in Research.

Reporting on sex and gender	N/A
Population characteristics	N/A
Recruitment	N/A
Ethics oversight	N/A

Note that full information on the approval of the study protocol must also be provided in the manuscript.

Field-specific reporting

Please select the one below that is the best fit for your research. If you are not sure, read the appropriate sections before making your selection.

☒ Life sciences ☐ Behavioural & social sciences ☐ Ecological, evolutionary & environmental sciences

For a reference copy of the document with all sections, see nature.com/documents/nr-reporting-summary-flat.pdf

Life sciences study design

All studies must disclose on these points even when the disclosure is negative.

Sample size	Sample size was determined mostly by the convenience levels of sample data acquisition. For mouse experiments, sample size was limited to the availability of Hexb-/- mice (sample size of other groups matched the number of Hexb-/- mice).
Data exclusions	For mouse behavioral experiments, the highest and lowest values for each mouse/each test was discarded. For all other experiments, no data exclusion was performed.
Replication	All experiments and data described in the study were repeated for 3-5 biologically independent repeats and all data were confirmed to be reproducible.
Randomization	Individual cells in images were randomly chosen for analysis, except excluding cells that were apparently abnormal by shape (double/splitted nucleus, apoptotic, aggregated). For other experiments, all individual samples were collected for analysis.
Blinding	For all image analyses, researchers quantifying images were blind to the sample group of images. For mouse behavioral tests, tester was blind to the group of the mice. For other experiments, blinding was not performed because no data point was excluded or selected (for STB and HBSS-treated cell death, numbers were read by photometer; for flow cytometry, numbers were counted by machine).

Reporting for specific materials, systems and methods

We require information from authors about some types of materials, experimental systems and methods used in many studies. Here, indicate whether each material, system or method listed is relevant to your study. If you are not sure if a list item applies to your research, read the appropriate section before selecting a response.

Materials & experimental systems

n/a	Involved in the study	
☐	☒	Antibodies
☐	☒	Eukaryotic cell lines
☒	☐	Palaeontology and archaeology
☐	☒	Animals and other organisms
☒	☐	Clinical data
☒	☐	Dual use research of concern

Methods

n/a	Involved in the study	
☒	☐	ChIP-seq
☒	☐	Flow cytometry
☒	☐	MRI-based neuroimaging

Antibodies

Antibodies used	LAMP1 (CST 9091S (clone D2D11) and DSHB H4A3), SNX2 (BD Biosciences, 611308, clone 13), SNX5 (abcam, ab5983, polyclonal), SNX8 (Life Span Biosciences, LS-C172487, clone 4F8), SNX9 (proteintech, 15721-1-AP, polyclonal), EEA1 (abcam, ab2900, polyclonal),

	GM130 (abcam, ab52649, polyclonal), RAB7 (abcam, ab137029, EPR7589), RAB5 (abcam, ab109534, EPR5438), Perilipin 2 (CST 95109S, clone E6G6M), Complex II (abcam, ab109865, clone 4H12BG12AG2), GLA (Abcam, ab168341, clone EP5828(2)), HEXA (Proteintech, 11317-1-AP), NPC1 (Novus, NB400-148), NeuN (Abcam, ab177487, clone EPR12763), GM2 (Abcam, ab23942), GFP (Santa Cruz, sc9996, clone B2), FLAG (Sigma F3165, clone M2), gamma-Tubulin (Sigma T5326, clone GTU-88), GAPDH (Sigma G8795)
Validation	LAMP1 antibody was validated by Cell Signaling Technology (https://www.cellsignal.cn/products/primary-antibodies/lamp1-d2d11-xp-rabbit-mab/9091) and previously (PMID: 23993788); SNX2, SNX8, GLA, HEXA, NPC1 antibodies were validated by KO cell lines in figures S1C and S4C; SNX5 antibody was validated previously (PMID: 33328639), SNX9 antibody was validated previously (PMID: 27655699), EEA1 antibody was validated previously (PMID: 8798641), GM130 antibody was validated previously (PMID: 30816200), RAB7 antibody was validated previously (PMID: 33846297), RAB5 antibody was validated previously (PMID: 33238112), Perilipin 2 antibody was validated by CST (https://www.cellsignal.cn/products/primary-antibodies/perilipin-2-e6g6m-rabbit-mab/95109), Complex II antibody was validated previously (PMID: 16120479), NeuN antibody was validated previously (PMID: 29752725), GM2 antibody was validated by Abcam; GFP antibody was validated by Santa Cruz (https://www.scbt.com/p/gfp-antibody-b-2); FLAG, gamma-tubulin and GAPDH antibodies were validated by Sigma Aldrich (https://www.sigmaaldrich.com/HK/zh/product/sigma/f3165; https://www.sigmaaldrich.com/HK/zh/product/sigma/t5326; https://www.sigmaaldrich.com/HK/zh/product/sigma/g8795).

Eukaryotic cell lines

Policy information about cell lines and Sex and Gender in Research

Cell line source(s)	HeLa cells were originally acquired from ATCC; mouse primary fibroblasts were acquired from BL6 WT and Hexb-/- p0 mice without record on sex.
Authentication	None were authenticated
Mycoplasma contamination	cell lines were not tested for mycoplasma contamination but were all treated with mycoplasma cleaning regent before used for experiments
Commonly misidentified lines (See ICLAC register)	cell lines used in the study were not listed as commonly misidentified lines

Animals and other research organisms

Policy information about studies involving animals; ARRIVE guidelines recommended for reporting animal research, and Sex and Gender in Research

Laboratory animals	BL6 WT and Hexb-/- mice were used in the study at ages 0-3 months.
Wild animals	The study did not involve wild animals.
Reporting on sex	sex was not considered in the study, as lysosome storage disease phenotypes were not reported to be sex-associated.
Field-collected samples	The study did not involve samples collected from the field.
Ethics oversight	Animal protocols used in the study were supervised by the Laboratory Animal Center of Zhejiang University.

Note that full information on the approval of the study protocol must also be provided in the manuscript.
